# Comparing bone shape models from deep learning processing of magnetic resonance imaging to computed tomography-based models

**DOI:** 10.1016/j.jseint.2023.05.008

**Published:** 2023-05-26

**Authors:** Victoria Wong, Francesco Calivá, Favian Su, Valentina Pedoia, Drew Lansdown

**Affiliations:** a Center for Intelligent Imaging, Radiology and Biomedical Imaging, University of California San Francisco, San Francisco, CA, USA; b Department of Orthopaedic Surgery, University of California San Francisco, San Francisco, CA, USA

**Keywords:** Artificial intelligence, Deep learning, Machine learning, Scapula, MRI, CT

## Abstract

**Background:**

The purpose of this study was to develop a deep learning approach to automatically segment the scapular bone on magnetic resonance imaging (MRI) images and to compare the accuracy of these three-dimensional (3D) models with that of 3D computed tomography (CT).

**Methods:**

Fifty-five patients with high-resolution 3D fat-saturated T2 MRI were retrospectively identified. The underlying pathology included rotator cuff tendinopathy and tears, shoulder instability, and impingement. Two experienced musculoskeletal researchers manually segmented the scapular bone. Five cross-validation training and validation splits were generated to independently train two-dimensional (2D) and 3D models using a convolutional neural network approach. Model performance was evaluated using the Dice similarity coefficient (DSC). All models with DSC > 0.70 were ensembled and used for the test set, which consisted of four patients with matching high-resolution MRI and CT scans. Clinically relevant glenoid measurements, including glenoid height, width, and retroversion, were calculated for two of the patients. Paired *t*-tests and Wilcoxon signed-rank tests were used to compare the DSC of the models.

**Results:**

The 2D and 3D models achieved a best DSC of 0.86 and 0.82, respectively, with no significant difference observed. Augmentation of imaging data significantly improved 3D but not 2D model performance. In comparing clinical measurements of 3D MRI and CT, there was a mean difference ranging from 1.29 mm to 3.46 mm and 0.05° to 7.47°.

**Conclusion:**

We have presented a fully automatic, deep learning-based strategy for extracting scapular shape from a high-resolution MRI scan. Further developments of this technology have the potential to allow for surgeons to obtain all clinically relevant information from MRI scans and reduce the need for multiple imaging studies for patients with shoulder pathology.

Shoulder bone morphology affects surgical outcomes for multiple conditions, such as shoulder instability, rotator cuff arthropathy, and osteoarthritis. The current gold standard for evaluation of shoulder bone morphology is three-dimensional (3D) computed tomography (CT) reconstructions as it provides accurate quantification of glenohumeral spatial relationships, bone loss, and wear patterns.^2^ However, CT scans are limited in their ability to evaluate soft tissue and expose patients to ionizing radiation. Magnetic resonance imaging (MRI) is frequently ordered in the setting of shoulder instability and arthropathy and provides superior soft tissue detail compared to CT. The utilization of both MRI and CT scans has been increasing recently as there is improved recognition regarding the complimentary information of bony and soft tissue structures.^21^ Ideally, all necessary information for surgical planning should be available from a single biomedical image to increase patient safety and convenience.

3D osseous reconstructions of the shoulder from MRI data may be an equally effective tool as CT in the evaluation of glenohumeral bone loss.^5^ However, these reconstructions often require either specialized sequences or extensive manual postprocessing steps. Manual segmentation of MR images can allow for 3D bony reconstructions, though this process is time-consuming, cumbersome, and not realistic to apply in clinical care. Prior semi-automated segmentations^20^^,^^19^^,^^9^ have been shown to increase efficiency by at least 3.5-fold while maintaining high accuracy and inter-annotator agreement. Despite these advances, the postprocessing time typically takes over 25 minutes. Additionally, prior reports have described automatic bony segmentation of the scapula, though these have typically required specific sequences and postprocessing.^12^ These requirements of extensive manual processing, specific sequences, or a need for a trained technologist have precluded the routine use of 3D MRI reconstructions in the clinical setting.

One proposed solution to improve the efficiency of 3D MRI bone segmentations is with the use of machine learning. Machine learning aims to recognize data patterns and trends to make informed decisions.^25^ Models learn through training, and performance is measured, and ideally improved, over time and repetition. Machine learning can be implemented to solve various tasks, such as classification, regression, detection, and image segmentation.^25^ Deep learning (DL), which is a branch of machine learning and artificial intelligence, has recently emerged as a strong tool for image analysis in medicine.^14^ However, because of the limited availability of suitable biomedical images, augmentation techniques are often used to artificially increase the size of data sets used to train DL models in radiology.^7^

The purpose of this study is to formulate a DL approach to automatically segment the scapular bone from MR images. We aim to compare the performance of models trained with 1) original versus augmented data, 2) axial versus coronal versus sagittal planes (for two-dimensional [2D] only), and 3) 2D versus 3D models. Additionally, we aim to evaluate our model’s performance to that of CT reconstructions, which are the current clinical gold standard. We hypothesize that the augmented data will improve model performance and that the 3D models will outperform the 2D models.

## Methods

### Patient identification

**Figure 1:**
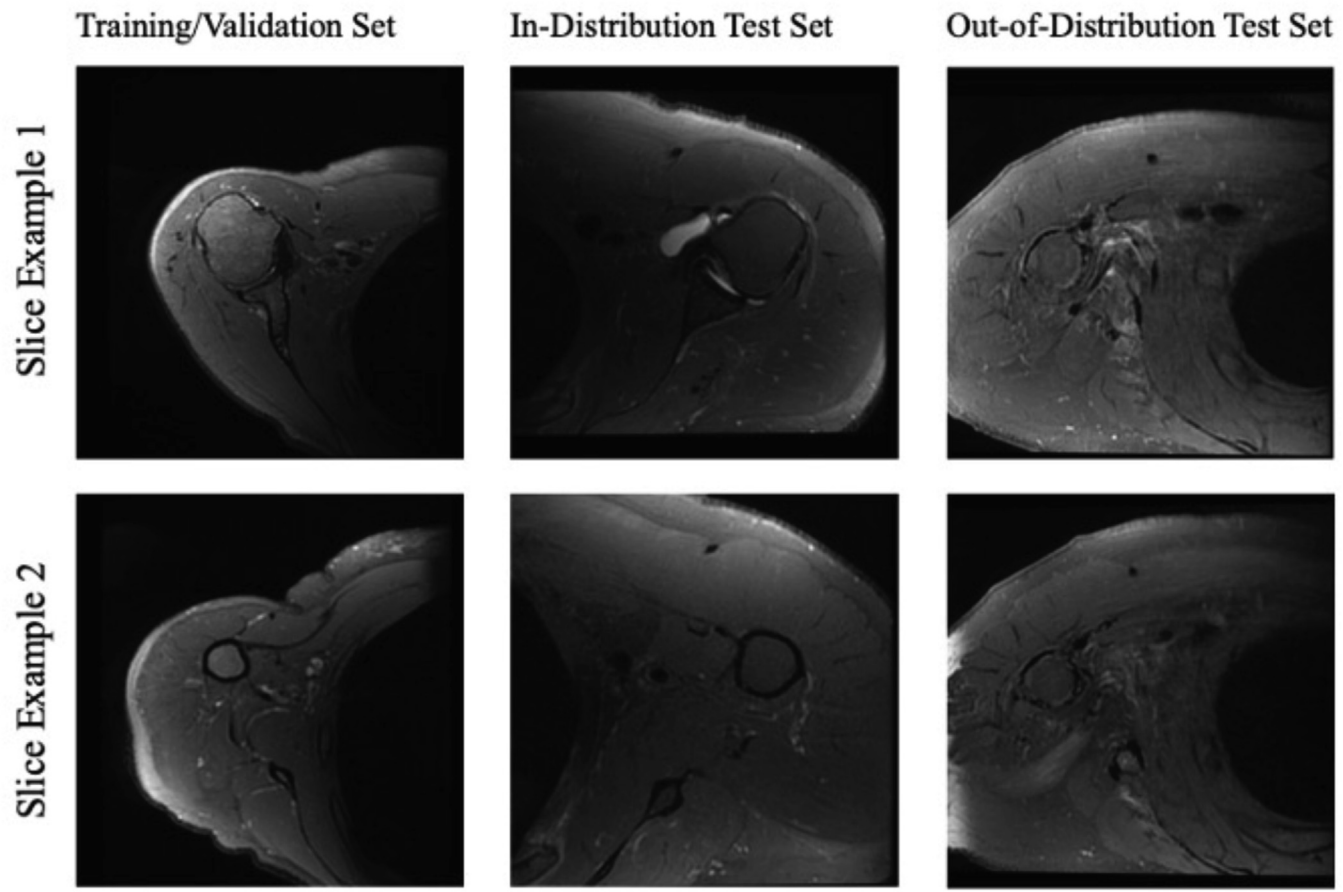
Example slices of MRIs used in this study. *MRI*, magnetic resonance imaging.

Fifty-five patients (mean age 51 years, range, 24-75) with high-resolution shoulder MRI scans were identified through the Picture Archiving and Communication System (PACS) (Table I) (Fig. 1). Diagnoses for these patients included rotator cuff tear or tendinopathy, shoulder instability, and impingement. Inclusion criteria was a shoulder MRI with a high-resolution axial sequence available (Table II) and performed between September 2018 and October 2020. Exclusion criteria included poor image quality, presence of metal artifact, fracture, suspected infection, or tumor. All procedures were approved by our institutional review board.

**Table I:** Patient demographics.

Age (y)	Mean: 51.5, range: 24-75
Sex	20 female, 35 male
Diagnosis	Rotator cuff injury (38), instability (9), impingement (5), tendinosis (3)
Laterality	29 left, 26 right
BMI (kg/m^2^)	Mean: 27.1, range: 18.0-40.5

*BMI*, body mass index.

**Table II:** MRI acquisition parameters.

Imaging parameter	Training/Validation set
Labeled data	55
Plane	Axial
Magnetic field strength (T)	3
Sequence	3D high-resolution fat-saturated (FS), T2-weighted
Pixel size (mm)	0.375 × 0.375
Slice thickness (mm)	1
Space between slices (mm)	0.5
Echo time (ms)	47.9
Repetition time (ms)	1352
Resolution	512 × 512
Flip angle (degrees)	90

*MRI*, magnetic resonance imaging; *3D*, three-dimensional.

### Data preparation

Manual segmentations of the scapula were performed to train and validate the DL model. Scans were manually segmented using an in-house program.^6^ Segmentation was performed by two experienced researchers. Two scans were manually segmented two additional times to account for interpersonal and intrapersonal reliability. Reliability was measured using Dice similarity coefficients (DSCs). The intra-rater and inter-rater reliability, respectively, were DSC = 0.91 and 0.85.

To decrease the computation load and allow training of a 3D model without resizing the image, each volume was cropped to exclude the first 112 pixels in both columns and rows. The volumes were sliced by selecting every other slice, starting from the first or the second slice. This cropping value ensured no loss of bone or scapula-related details, and the 3D volume size after this procedure was two volumes of 400 × 400 × 124 (initially one volume of 512 × 512 × 248).

Cropped images were then augmented with random affine, elastic deforms, Gaussian, and/or noise transformations.^24^ Each original image had a 30% chance of undergoing any of the listed augmentations, and each was augmented eight times.

### Data splitting

Patients were then divided into five cross-validation sets using the K-folds cross-validator. This method splits the data set into k consecutive folds (without shuffling by default). Each fold is then used once as a validation while the k-1 remaining folds form the training set.

### Network details

To begin training, data were first normalized. In the training set for 3D models, normalization was done using a random value within the range of maximum volume value ± 1000. This normalization accounted for potential coil artifacts, which would strongly alter image intensity values. As a result, data were inherently further augmented. In the validation set, normalization was done by dividing by the maximum volume value. Additional division by the 85th percentile proved helpful to account for potential outlier values, as shown in previous studies.^4^^,^^11^ In the training set for 2D models, a similar normalization was done, using instead a value within the range of maximum volume value ± 500, as this resulted in a more stable outcome relative to 1000.

To choose a network size for both 2D and 3D, V-Net models of different width and depth were tested. Models that were too small were not successful in fitting the training set, whereas models that were too large quickly overfit the data. Satisfactory training was obtained with the V-Net characteristics as shown in Table III.

**Table III:** V-Net characteristics used for satisfactory training of 2D and 3D models.

	2D	3D
Architecture	- Number of channels: 16	- Number of channels: 12
	- Number of levels: 2	- Number of levels: 4
	- Number of convolutions: [4, 4]	- Number of convolutions: [1, 3, 4, 3]
	- Bottom convolutions: 2	- Bottom convolutions: 4
Learning rate	0.0005	0.00001

*2D*, two-dimensional; *3D*, three-dimensional.

A convolution is when two sets of information are merged to form a new function.

### Training details

Cross entropy, Dice loss, and weighted combinations thereof were tested. Ultimately, the best-performing function was the negative logarithmic dice loss:

$$F=-\mathrm{log10}\frac{2\left|p\cdot \hat{y}\right|}{\left|p\right|+\left|\hat{y}\right|}$$

with p the predicted segmentation probability map and $\hat{y}$ the segmentation ground truth mask.

2D and 3D models were trained for a maximum number of 100,000 iterations. However, the model was subject to early stopping if no improvements in the validation set were observed for 30 validation epochs. An epoch refers to one entire passing of data through the algorithm. A validation epoch was conducted every 90 training steps. Dropout with keep probability of 95% was also implemented and Adam optimizer was employed with standard internal parameters.

Ten 3D models were trained: 5 with original data (one for each cross-validation split) and 5 with data augmentation (one for each cross-validation split). Multiple 2D models were trained to separately perform segmentation on axial, sagittal, and coronal view. The 3D volumes were acquired axially and sliced accordingly to obtain sagittal and coronal views. Subsequently, data were processed on a per-slice basis. Also, for 2D models, multiple models were trained using the different cross-validation splits.

### Ensemble

After training and validating 40 total models, all models with DSC above 0.70 were ensembled. The ensemble was run on the test set to measure the final performance. The ensemble models were used to calculate glenoid measurements.

### Test set

**Figure 2:**
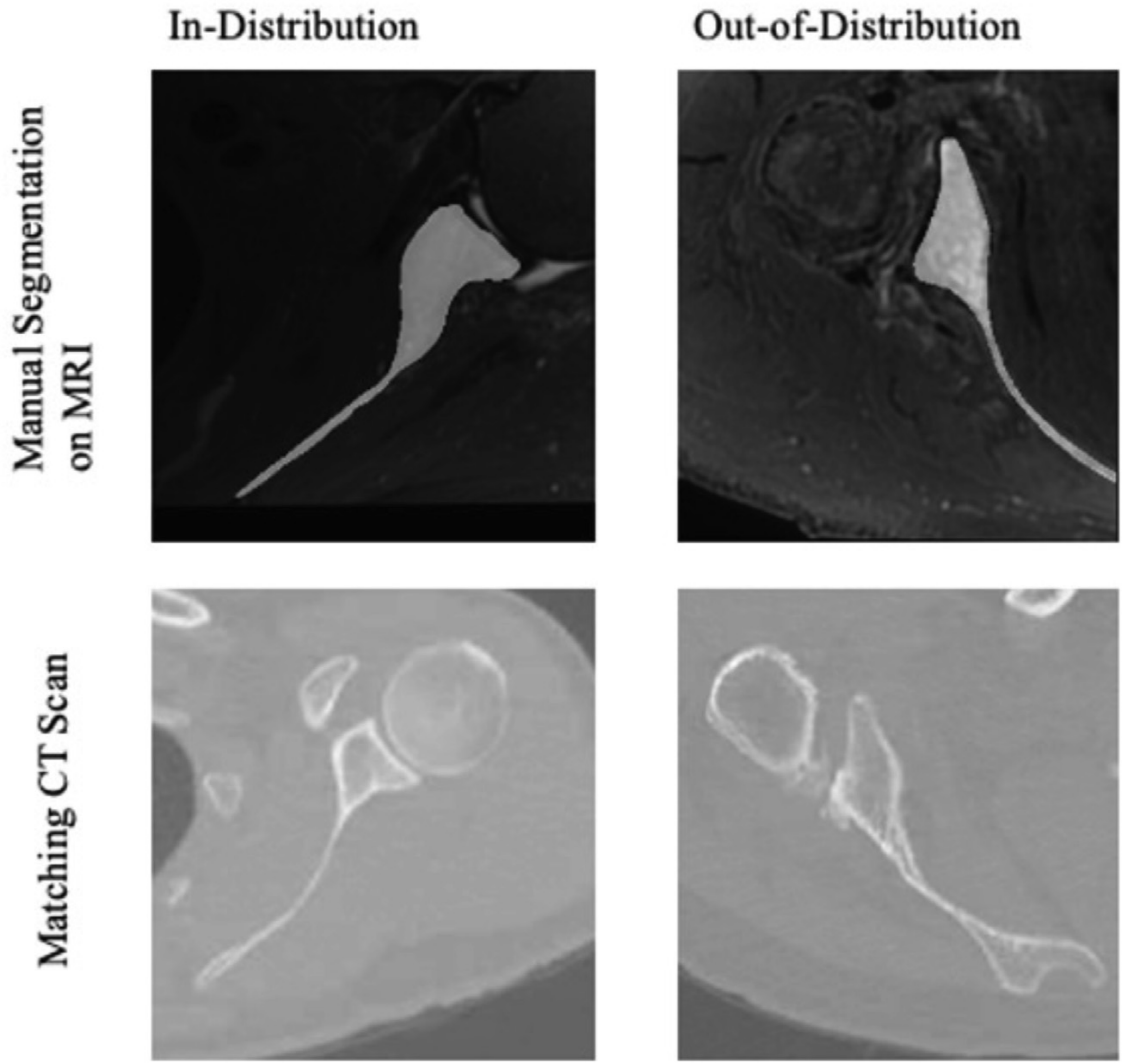
Manual segmentation overlayed on MRI and corresponding CT scans for in-distribution and out-of-distribution patients in our test set. *MRI*, magnetic resonance imaging; *CT*, computed tomography.

Four patients with matching high-resolution MRI and CT scans (slice thickness: 1.25 mm) were identified through the PACS system and comprise the test set (Fig. 2). Two of these patients’ MRIs were the same sequence as the MRIs used in the training set, thus comprising the in-distribution test set (ages 57 and 71, both male, cuff tear and arthritis diagnoses, average body mass index 31 kg/m^2^). The other two patients had different high-resolution MRI sequences to create our out-of-distribution test set (ages 61 and 76, both male, instability and cuff tear diagnoses, average body mass index 31 kg/m^2^).

### Important glenoid measurements

**Figure 3:**
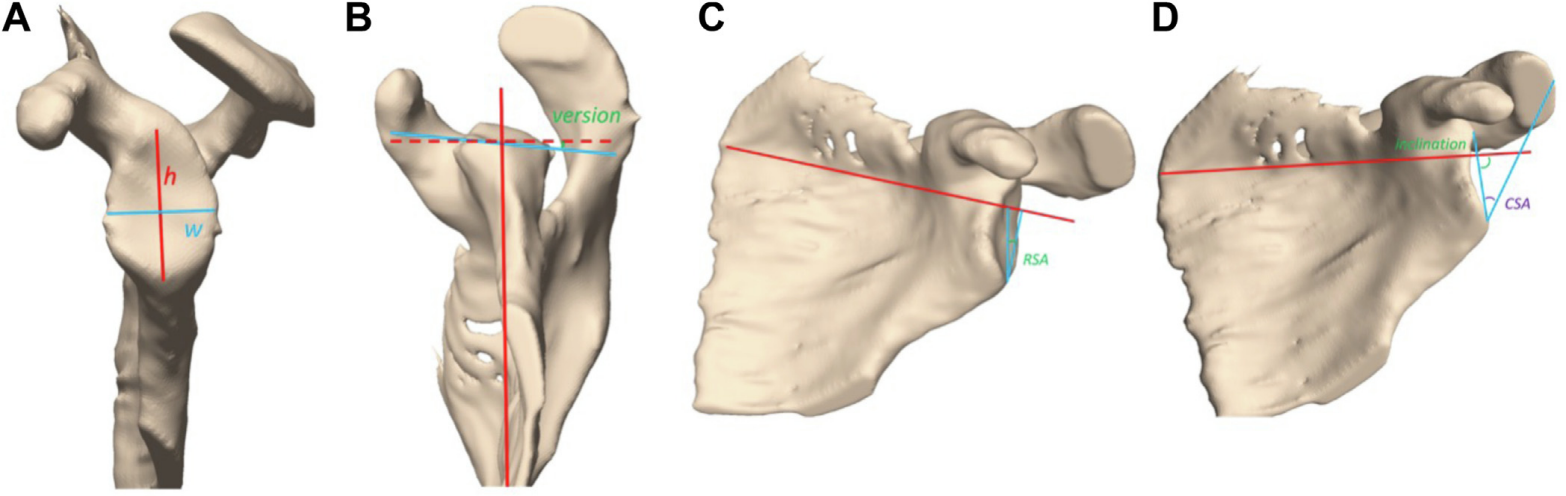
Clinically important shoulder measurements shown on 3D scapula reconstructions from MRI. (**A**) Glenoid height and width. (**B**) Glenoid version. (**C**) Reverse shoulder arthroplasty angle. (**D**) Glenoid inclination angle and critical shoulder angle. *MRI*, magnetic resonance imaging; *3D*, three-dimensional.

In addition to DSC, we obtained glenoid measurements from our automatic models to better assess model performance for clinically relevant features. These measurements included glenoid height, width, retroversion, reverse shoulder arthroplasty angle (RSA angle), inclination angle, and critical shoulder angle. Glenoid height (Fig. 3*A*) and width (Fig. 3*A*) are important for assessing bone size and glenoid bone loss.^5^ Glenoid retroversion (Fig. 3*B*) is the angle between the line from the anterior to the posterior glenoid edge and the line from the glenoid center to the medial edge of the scapula.^22^ RSA angle (Fig. 3*C*) is the angle between the line connecting the inferior glenoid and lateral supraspinatus fossa and the perpendicular of a tangential line through the floor of the supraspinatus fossa.^3^ Glenoid inclination angle (Fig. 3*D*) is defined as the angle from the most inferior aspect of the glenoid to the most superior aspect of the glenoid and a line tangential to the floor of the supraspinatus fossa.^10^^,^^13^ Critical shoulder angle (Fig. 3*D*) is defined as the angle between the line between the inferior and superior glenoid and the line from the inferior glenoid to lateral acromion.^18^

### Statistical analysis

Manual segmentations were used to evaluate the performance of the 2D and 3D MRI models through DSC. Statistical tests were utilized to determine: (1) if models with original or augmented data performed better; (2) which 2D plane (axial, coronal, or sagittal) performed the best; and (3) if 2D or 3D models performed better.

Shapiro-Wilk tests were first used to determine if the DSC of model performances were normally distributed. Paired Student *t*-tests were used for normally distributed data and Wilcoxon signed-rank tests were used for nonparametric tests. 3D MRI and CT model measurements were compared by calculating the mean difference. Significance was defined as *P* < .05. All statistical analyses were performed using Stata 16.1 (StataCorp, College Station, TX, USA).

## Results

We trained and validated a total of 40 different models to automatically segment the scapular bone from MRI. Mean values and standard deviations are reported in Table IV.

**Table IV:** Model performance results.

Model	Mean dice	Standard deviation
3D original data	0.66	0.230
3D augmented data	0.80	0.028
2D axial—original data	0.79	0.045
2D axial—augmented data	0.80	0.025
2D coronal—original data	0.80	0.035
2D coronal—augmented data	0.81	0.033
2D sagittal—original data	0.78	0.069
2D augmented data	0.80	0.074

*2D*, two-dimensional; *3D*, three-dimensional.

Results from statistical analysis are reported in Table V, Table VI, Table VII. The 3D model trained with augmented data outperformed the 3D model trained with original data (*P* = .043). There were no significant differences in the performances of the 2D models trained with augmented versus original data for all three views (axial, coronal, or sagittal) (*P* = .657, .345, .594). There were no significant differences in model performance for imaging plane (axial, coronal, or sagittal) (*P* = .700). There was also no significant difference in the performance of 2D and 3D augmented models (*P* = .893).

**Table V:** Statistical analysis of original versus augmented data.

Model	Null hypothesis	*P* value	Results
3D	No difference	.043 (Wilcoxon)	Augmented > original
2D axial	No difference	.657	No significance
2D coronal	No difference	.346 (Wilcoxon)	No significance
2D sagittal	No difference	.594	No significance

*2D*, two-dimensional; *3D*, three-dimensional.

**Table VI:** Analysis of variance (ANOVA) analysis of 2D planes.

Model	F value	*P* value	Results
Axial vs coronal vs sagittal	0.37	.700	No significance

*2D*, two-dimensional.

**Table VII:** Statistical analysis comparing our 3D augmented model and 2D axial model.

Model	Null hypothesis	*P* value	Results
3D augmented vs 2D axial augmented	No difference	.893 (Wilcoxon)	No significance
3D augmented vs 2D axial original	No difference	.500 (Wilcoxon)	No significance

*2D*, two-dimensional; *3D*, three-dimensional.

The best 2D model performance was by the augmented sagittal model validated on cross-validation set 1 (DSC = 0.86) (Table IV). The best 3D model performance was by the augmented model validated on cross-validation set 0 (DSC = 0.82) (Table IV). There was no significant difference between the best 2D and the best 3D models (*P* = .500) (Table V, Table VI, Table VII).

After ensembling all models with validation DSC greater than 0.70, 4 models were excluded (3D original data on cross-validation set 3, 3D original data on cross-validation set 4, 2D sagittal original data on cross-validation set 0, and 2D sagittal augmented data on cross-validation set 4). Ensemble performance was determined for the test set (Table VIII). In-distribution subjects performed substantially better than out-of-distribution subjects.

**Table VIII:** Performance of test set, separated by in- and out-of-distribution subjects.

	In-distribution		Out-of-distribution	
Patient number	056	057	058	059
DSC	0.89	0.81	0.24	0.58

*DSC*, dice similarity coefficient.

**Figure 4:**
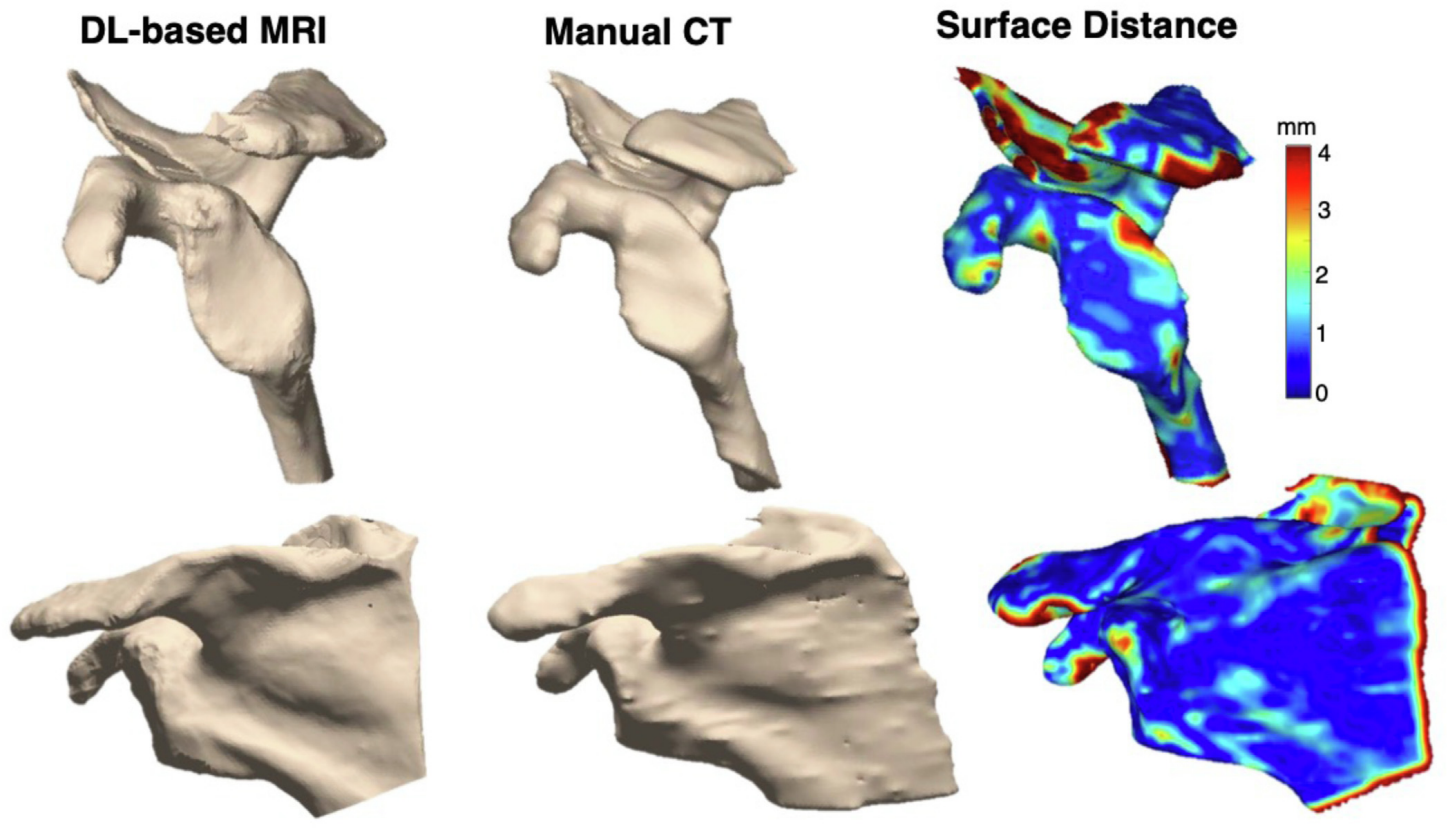
3D models of model vs manual performance with surface distance map to measure differences. *DL*, deep learning; *MRI*, magnetic resonance imaging; *3D*, three-dimensional; *CT*, computed tomography.

After constructing scapula models of our in-distribution test set using the ensemble, clinically important measurements from the model were compared to that of CT (Fig. 4) (Table IX). Mean differences for distances were between 1.29 mm and 3.46 mm, while mean differences for angular measurements were between 0.05° and 7.47°.

**Table IX:** Comparison of important glenoid measurements of our models and CT scans.

	MRI 056		CT 056		Mean difference	MRI 057		CT 057		Mean difference
	Trial 1	Trial 2	Trial 1	Trial 2		Trial 1	Trial 2	Trial 1	Trial 2	
Width (mm)	27.52	26.88	28.37	28.60	1.29	31.15	31.23	33.06	32.97	1.83
Height (mm)	43.05	40.94	39.12	37.96	3.46	48.89	46.47	46.70	45.78	1.44
Inclination angle (˚)	97.48	99.32	95.73	97.53	1.77	69.89	69.25	66.03	66.49	3.31
Retroversion (˚)	0.74	−0.50	4.69	2.08	3.27	32.10	35.50	29.39	31.45	3.38
Critical shoulder angle (˚)	30.45	31.63	27.43	29.21	2.72	27.11	26.65	27.20	26.66	0.05
RSA angle (˚)	7.87	11.30	10.74	12.93	2.28	14.18	15.65	16.33	17.30	1.90

*CT*, computed tomography; *MRI*, magnetic resonance imaging; *RSA*, reverse shoulder arthroplasty.

## Discussion

A DL strategy can allow for automatic scapular bone shape extraction from high-resolution MRI. We achieved Dice score coefficients as high as 0.86. Model performance was similar between 2D and 3D models, which was contrary to our hypothesis that 3D models would perform better. Augmentation of the imaging data did not improve model performance for the 2D models, but this approach was advantageous for 3D models. In comparing surface measurements between the MRI-based and CT-based reconstructions, we observed mean differences of MRI and CT measurements ranging from 0.05 to 7.47 degrees and 1.29 to 3.46 mm.

We trained both 2D and 3D convolutional neural networks (CNNs). 2D CNNs predict segmentation maps of single slices, whereas 3D CNNs utilize a volume to make predictions. Thus, the main difference between these approaches is that 3D models consider information from adjacent slices while 2D models do not. 3D models may often lead to better performance but require much more computational power. 2D models, while needing less computation resources, take more time to infer a 3D model from 2D slices.^17^

We had anticipated that models trained with augmented data would outperform those trained with only the original data set, though we found this to be only true for 3D approaches and not 2D approaches. In our experiment, data were augmented eight times to overcome the problem of limited data. Doing so increased the size, quality, and generalizability of our training set, thus obtaining more information from the same data. Our sample size was small, due to the time-consuming nature of manually segmentation and limited number of clinically available scans. Quality and generalizability of data are important to prevent overfitting of the model for the specific data it was trained on and ensure it is applicable to new data. Other approaches to data augmentation have been studied. Most augmentation steps, including ours, deploy transformations to rotate, reflect, or deform images.^23^^,^^8^^,^^16^ Eaton-Rosen et al suggest a mix-up technique that utilizes linear combination of two training data to create images that are quite different from training images. They found that it can improve performances of machine learning tasks.^7^ One possible explanation for the difference in augmentation performance for 3D vs 2D models may be that our augmentation process was not enough to offer extra noise to the training model. In future experiments, we may increase the chances and degree of random augmentation.

While the DSC observed here are high, they are lower than those reported in other bone shape models. A similar study that segmented bone from 3D knee MRIs achieved a DSC of 0.97.^1^ The scapula bone presents several challenges to automatic segmentation due to its complex shape. The main culprit of our lower DSC may be the thinness of the scapular spine, which presented challenges in both manual segmentation and model training. Fortunately, the scapular spine is of less clinical significance than the glenoid. The clinically relevant measurements support this interpretation as there are minimal differences in these regions. The comparisons between MRI and CT models also show differences through the glenoid face, which would be most important for clinical use.

There have been other approaches to constructing bone models from MRIs, such as the use of specific MRI sequences to enhance bone contrast and allow for 3D models to be rendered. One example of a specific sequence is 3D Zero Echo Time (ZTE) MRI.^15^ This approach proved to accurately measure glenoid bone loss in both cadavers and patients, demonstrating potential clinical applications. Another MRI sequence that allowed for similar results is Dixon fat-water separation sequence.^12^ Both are comparable to CT scans and allow for preoperative planning to be conducted with only one biomedical image. These approaches, however, are limited in a few senses. First, the sequence must be known and requested before image acquisition as conventional MRIs cannot be converted later. Thus, patients who are evaluated with pre-existing MRIs may have to acquire a new image. Additionally, these sequences need specialized imaging equipment that may not be easily accessible to all patient populations while the postprocessing described here could be applied more widely. Our DL approach was able to produce a 3D bone from high-resolution MRIs, needing only 2-3 seconds of processing time. This method is not only fast enough for clinical application but also overcomes the limitation of needing a specific sequence prior to image acquisition as DL can be applied to pre-existing scans with minimal postprocessing.

The findings of this study should be interpreted with an understanding of its limitations. First, the sample size of patients for testing is small due to the availability of matching CT and MRI with a high-resolution sequence. Furthermore, most patients included in this study had a diagnosis of rotator cuff injury and instability. Measurements of bone shape may vary more for patients with severe arthritis, fracture, postsurgical changes, or other artifacts that may limit the ability of automatic model development. Future studies would need to include patients with severe osteoarthritis, bone loss, and deformity to expand its clinical applications. We also utilized a high-resolution MRI sequence. Future efforts will work to translate this approach to more standard MRI sequences.

## Conclusion

We have presented a fully automatic, DL-based strategy for extracting scapular shape from a high-resolution MRI scan. Future work will expand this methodology to patients with a broader range of pathology. This image processing technology has the potential to greatly improve the diagnostic and preoperative planning process for patients with shoulder pathology.

## Disclaimers

Funding: Research reported in this publication was supported by NIA grant number R38AG070171. Its contents are solely the responsibility of the authors and do not necessarily represent the official views of the NIA.

Conflicts of interest: The authors, their immediate families, and any research foundation with which they are affiliated have not received any financial payments or other benefits from any commercial entity related to the subject of this article.
